# High molecular pyrogens present in plant extracts interfere with examinations of their immunomodulatory properties in vitro

**DOI:** 10.1038/s41598-020-79579-2

**Published:** 2021-01-12

**Authors:** Aleksandra Kruk, Jakub P. Piwowarski, Karolina A. Pawłowska, Dominik Popowski, Sebastian Granica

**Affiliations:** 1 Department of Pharmacognosy and Molecular Basis of Phytotherapy, Centre for Preclinical Studies, Faculty of Pharmacy With the Laboratory Medicine Division, Medical University of Warsaw, ul. Banacha 1, 02-097 Warsaw, Poland; 2 Centre for Preclinical Studies, Medical University of Warsaw, ul. Banacha 1b, 02-097 Warsaw, Poland

**Keywords:** Drug discovery, Cytokines

## Abstract

The widely accepted strategy to justify the use of medicinal plant extracts in diseases with inflammatory background is their examination on in vitro models using immune cells. It is also a key initial step of research for active principles, which could be then isolated and tested on more advanced models, becoming new pharmacologically active lead molecules. The crucial aspect which has not been so far addressed in this context, is the presence of pyrogens in plant preparations. The aim of this study was the examination of pyrogens interference with in vitro evaluation of anti-inflammatory activity of plant extracts using human primary neutrophils model together with introduction of effective method of interfering factors elimination. The obtained results showed that chosen plant extracts contained pyrogens, which were responsible for concentration-dependent stimulation of pro-inflammatory cytokines production by human neutrophils in vitro in the same extent as LPS did. The ultrafiltration method was successfully applied for pyrogens elimination, which effectiveness was confirmed using LAL test. The determined interference of pyrogens implies the necessity of their consideration and removal when in vitro studies include direct addition of plant extracts to the cell culture, what can be obtained by ultrafiltration, which does not affect extract composition.

## Introduction

The widely accepted strategy to justify the use of medicinal plant extracts in diseases with the inflammatory background is their examination on in vitro models of immune cells responsible for the progression of inflammation^1–4^. It also the first step of a search for active principles which could be then isolated and tested on more advanced ex vivo and in vivo models. The commonly raised limitation of this strategy is the question regarding the bioavailability of plant extract constituents. This drawback can be however addressed if we consider in vitro examination of plant extracts as an initial step before conducting in vivo studies or as a screening strategy which leads to a selection of the most effective extraction procedure and active composition followed by bio-guided fractionation and subsequent isolation of active principles which will be next subjected for further pharmacological evaluations^5^.

The crucial aspect which so far has not been addressed in the in vitro studies of plant extracts is the possible presence of pyrogenic factors in tested preparations deriving from bacteria residing on raw plant materials. Their interference with in vitro assays, especially targeted on the modulation of the inflammatory response can occur particularly detrimental by leading to false-positive or false-negative results. Bacterial endotoxins, such as lipopolysaccharide, are the most common pyrogens, which if present even at a very low concentration in tested extracts can potentially interfere with examined inflammation processes leading to disrupted results.

The inflammation is a complex process of an immune system response to the pathogenic agents infecting the organism. The aim of the inflammatory response is the prevention and elimination of the infecting agents from the organism, reparation of damaged tissues and prevention of further disease development. Although many factors can trigger the inflammation, the mechanisms of the immune system response are generally common for all of them. The widely applied therapies in diseases with excessive inflammatory response include non-steroidal anti-inflammatory drugs, which due to the inhibition of COX-2 activity lead to attenuation of the processes orchestrated by immune cells. Despite high efficiency, the use of these drugs is associated with serious adverse effects including damage to the gastric mucosa as well as kidney and liver disorders. The consequence of this limitation is ongoing research on novel agents, which could modulate the inflammatory response in an alternative mechanistic mode, that it will be still effective towards pathogens, but will not cause the significant damage to the host tissues^6,7^. Extensive research has been done in a field of natural products using a screening-based approach for identification of active principles which could serve as potential drugs in inflammation-associated diseases^8^. Plant materials were shown to be a source of potential drugs which can actively modulate the inflammatory response. COX-1 inhibitor—salicylic acid isolated from *Filipendula ulmaria* can serve as a good example of an active principle isolated from medicinal plants traditionally applied in the treatment of diseases with inflammatory background, which has been modified to improve its pharmacokinetic properties^9,10^.

Medicinal plant materials are currently seen as an important source of compounds, which can serve as lead molecules in the development of novel anti-inflammatory agents. Plants have yielded a large number of species with acclaimed anti-inflammatory effects. For centuries inflammation-associated diseases were treated with orally applied plant infusions, decoctions and intracts. For this purpose, both plant fragments, such as flowers, leaves and seeds, and even whole parts thereof were used^11,12^. The emerging development of natural product formulations utilizing the unique anti-inflammatory compounds such as polyphenols, polysaccharides, alkaloids, terpenes, fatty acids, proteins and a wide array of other bioactive components has shown notable successes^13,14^.

Neutrophils contribute to the first line of the immune response towards all sort of exogenous factors and pathogens and were shown to play a crucial role in all diseases with the inflammatory background. They are the type of granulocytes that constitute the majority of white blood cells in organisms of mammals^15^. Neutrophils are phagocyte-type cells, they are part of the innate immune system. Their function is facilitated by the expression of TLR family members, allowing the recognition of a wide repertoire of PAMPs and thus triggering the response to invading pathogens. TLR4 is the most studied of all of the neutrophil TLRs, mediating responses to Gram-negative bacteria, which recognises the lipid A component of lipopolysaccharide^16^. The neutrophil activation leads to infiltration through the blood vessels and interstitial tissue to the inflamed site, following chemical signals in the chemotaxis process, where they destroy the pathogens^17,18^.

Neutrophils use three mechanisms to eliminate the pathogens: phagocytosis, degranulation and NETs^19^. In chemotaxis process, the migration of neutrophils in the direction of infection is orchestrated by the exogenous (PAMPs) and endogenous factors such as chemokines IL-8, cytokines (IL-1β or TNF-α) or other DAMPS, which are produced by cells in response to injury or infection^20^. Overstimulation of neutrophils caused by a variety of stimuli could lead to chronic, non-infectious inflammation^12^. Above properties of neutrophils and their easy accessibility as primary immune cells isolated from human peripheral blood, constitute their usefulness in in vitro experiments focused on the examination of inflammation-associated processes.

The aim of this study was an examination of the impact of infusions and intracts prepared from selected plant materials on apoptosis and cytokines release by human primary neutrophils. The observed significant induction of inflammatory response led to the identification of extract-derived pyrogenic factors, which interfered with applied in vitro model. The effective method of pyrogens' elimination was elaborated.

## Results

### Influence of extracts on neutrophils’ viability and apoptosis

**Figure 1:**
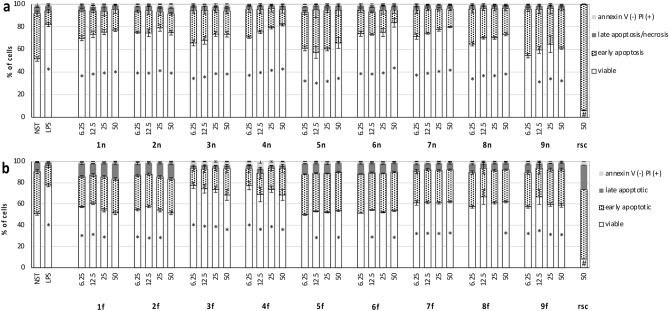
Effect of native plant extracts **1n**–**9n** (**a**) and extracts after ultrafiltration **1f.**–**9f.** (**b**) at the concentration 6.25, 12.5, 25 and 50 μg/mL on apoptosis and necrosis of neutrophils. Roscovitine (rsc) at the concentration of 50 μM was used as a positive control. Data were expressed as mean ± SEM of three separate experiments conducted in triplicate. The experiments were performed with cells isolated from three independent donors. Statistical significance *p < 0.05, ^#^p < 0.05 versus non-stimulated control—NST (Dunnett’s post-hoc test).

None of the tested extracts expressed cytotoxic effect on neutrophils (Fig. 1a). Most of the tested extracts inhibited the naturally occurring apoptosis showing the percentage of viable cells comparable or higher than the non-stimulated control. The inhibition of apoptosis for extract **1n**–**4n** and **6n**–**7n** was similar to this observed for LPS, which is known to prevent the apoptosis in human neutrophils^21^. Mostly, the number of living cells increased with extracts concentrations, while the percentage of apoptotic cells simultaneously decreased. Roscovitine (a CDK inhibitor) used as positive control showed a high level of apoptotic cells (93.8 ± 0.3%) and low level of viable cells (6.2 ± 0.3%). Similarly, the extracts subjected to ultrafiltration showed no cytotoxic effect on neutrophils (Fig. 1b) However **1f.**, **2f.**, **6f.** and **7f.** were no longer preventing apoptosis as it was observed for respective native extracts. For **1f.**, **2f.** and **4f.**–**9f.**, the level of viable, early and late apoptotic cells was similar to the non-stimulated control. For **3f.** and **4f.** the percentage of viable cells was still higher than for non-stimulated control and was comparable with LPS-stimulated control. At the same time, a significant decrease in apoptotic cells was observed. These observations suggest that **3f.** and **4f.** contain agents, which prevent neutrophil apoptosis, being of different nature than the pyrogens eliminated during ultrafiltration.

### Effect on IL-8 production

**Figure 2:**
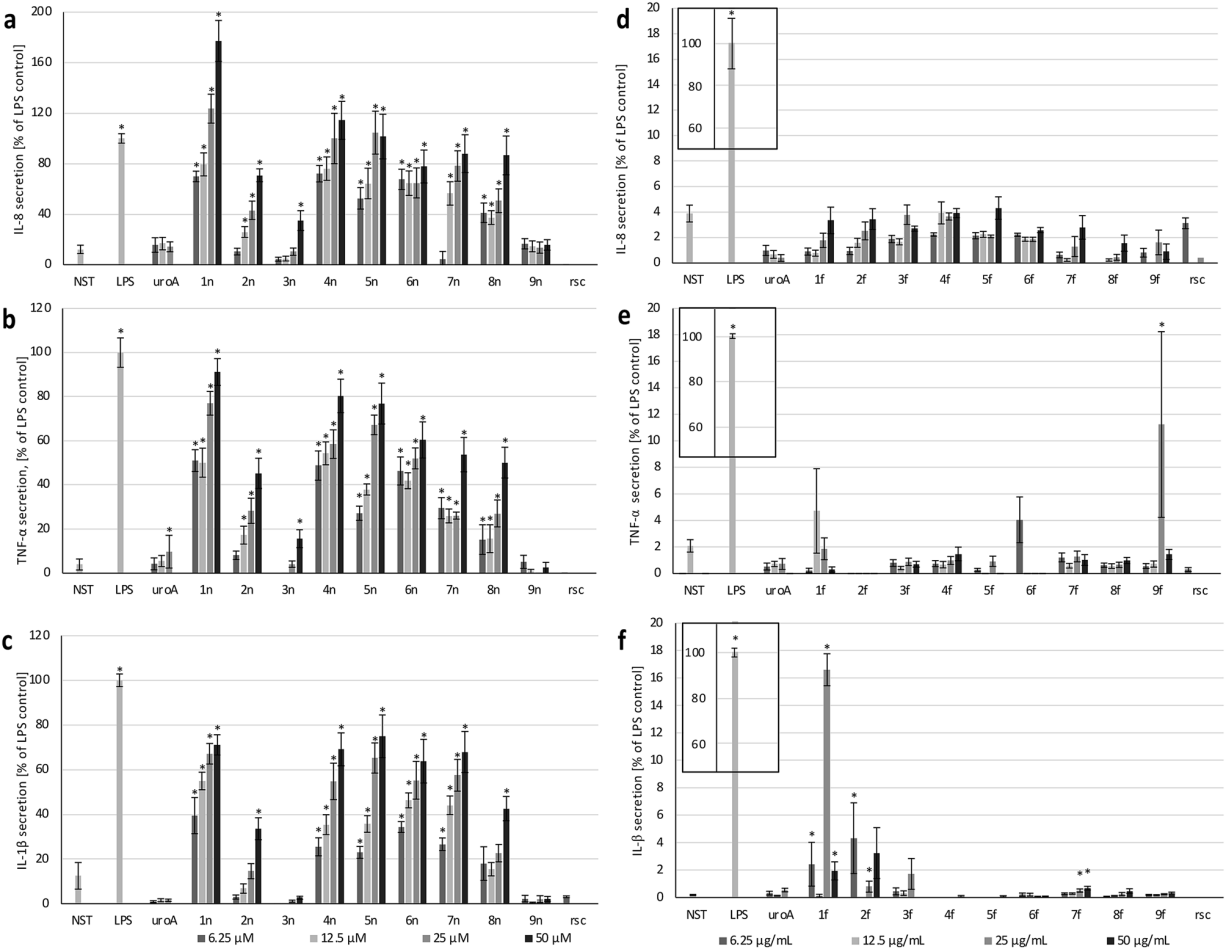
Effect of native plant extracts **1n**–**9n** (**a**–**c**) and extracts after ultrafiltration **1f.-9f.** (**d**–**f**) at the concentration 6.25, 12.5, 25 and 50 μg/mL 6.25, 12.5, 25 and 50 μg/mL on IL-8, TNF-α, IL-1β production by neutrophils. Urolithin A (uroA) at the concentration of 5, 10, 40 μM and roscovitine (rsc) at the concentration of 50 μM were used as a control. Data were expressed as mean ± SEM of three separate experiments conducted in triplicate. The experiments were performed with cells isolated from three independent donors. Statistical significance *p < 0.05 increase versus non-stimulated control (NST) (Dunnett’s post-hoc test).

All tested extracts were shown to induce IL-8 production (Fig. 2a). The exceptions were **2n** (at concentration 6.25 μg/mL), **3n** (6.25–25 μg/mL) and **9n** (6.25–50 μg/mL), which had a value comparable the non-stimulated control. Other extracts, at 6.25–12.50 (**1n**, **4n**, **5n**, **7n**) or 6.25–25.0 μg/mL (**6n**, **8n**) showed IL-8 secretion lower than stimulated control and highest than non-stimulated. The highest values were observed for extracts **1n**, **4n**, **5n** at 25–50 μg/mL, for which cytokine production was higher than for cells treated with LPS (100 ng/mL) (123.6 ± 11.5 and 177.2 ± 16.1% (**1n**), 100.0 ± 19.9 and 114.4 ± 15.1% (**4n**), 104.6 ± 16.9 and 101.49 ± 17.8% (**5n**) respectively). For all extracts except **6n** and **9n**, the stimulation of cytokine production increased with the concentration of the extracts. Urolithin A (a positive control known to be TLR4 signalling inhibitor^22^) showed results comparable to the non-stimulated control independently on the concentration whereas roscovitine due to induced cell apoptosis completely inhibited the production of this cytokine.

### Effect on TNF-α production

Extracts **1n** and **4n**–**8n** at 6.25–12.5 µg/mL showed stronger TNF-α production than the non-stimulated control (Fig. 2b). Additionally, at 25–50 µg/mL, the cytokine production increased to the level close to stimulated control ((76.9 ± 5.4 and 91.2 ± 6.0% for **1n**) or below (28.3 ± 5.7 and 45.2 ± 6.8%; 58.4 ± 6.5 and 80.4 ± 7.6%; 67.2 ± 4.4 and 76.9 ± 9.3%; 51.9 ± 4.8 and 60.3 ± 8.2%; 26 ± 1.7 and 53.6 ± 7.8%; 27.1 ± 6.1 and 50.0 ± 7.0% for **2n**, **4n–8n** respectively). The lowest TNF-α production was observed for **2n** (at 6.25 μg/mL), **3n** (at 6.25–25 μg/mL), **9n** (at 6.25–50 μg/mL) and was comparable to non-stimulated control. In most cases except **9n**, the induction of TNF-α production depended on the concentration of the extracts. For roscovitine and urolithin A results did not differ significantly from the non-stimulated control.

### Effect on IL-1β production

For **1n** and **4n**–**8n** at 6.25–12.5 µg/mL IL-1β production was higher than the non-stimulated control (Fig. 2c). Then, at 25 and/or 50 μg/mL the released cytokine values increased and finally reached 71.1 ± 4.5, 69.1 ± 7.4, 75.0 ± 9.6, 63.8 ± 9.8, 69.0 ± 9.2 and 42.6 ± 5.5% for **1n** and **4n**–**8n** respectively. For **2n** (at 6.25–12.5 µg/mL), **3n** (at 6.25–50 μg/mL) and **9n** (at 6.25–50 μg/mL) the cytokine production was lower than the non-stimulated control. All extracts except **9n** showed a dependence of IL-1β secretion on the concentration of the extracts. For urolithin A and roscovitine cytokine production was lower than for non-stimulated control.

### Effect of filtered extracts on cytokines' production

Ultrafiltration was shown to fully remove the inducing effect of extracts towards the production of IL-8, TNF-α (Fig. 2d,e). In the case of IL-1β production (Fig. 2f) extracts **4f.**–**8f.** lost their inducing activity due to conducted ultrafiltration. A different situation was observed for **1f.** and **2f.**, for which at concentrations 6.25, 25 and 50 µg/mL slight stimulation of cytokine production (2.4 ± 1.6, 16.6 ± 1.2, 1.9 ± 0.7% for **1f.** and 4.3 ± 2.6, 0.8 ± 0.4, 3.2 ± 1.8% for **2f.**) was determined. Urolithin and rosocovitine did not induce production of any of tested cytokines.

### Evaluation of endotoxins content in examined extracts

**Figure 3:**
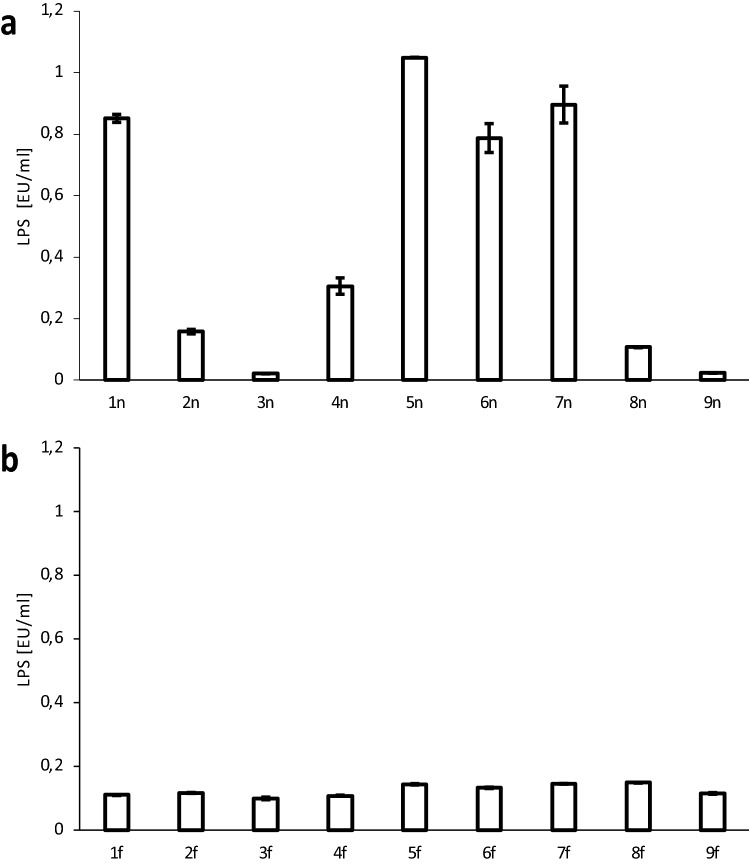
LPS content in native plant extracts **1n**–**9n** (**a**) and extracts after ultrafiltration **1f.**–**9f.** (**b**) at the concentration 50 μg/mL. Analytical sensitivity: 0.1 EU/mL.

To evaluate the content of pyrogenic factors LAL test was conducted for all extracts at the highest applied concentration of 50 μg/mL (Fig. 3a). The lowest LPS content was observed for **3n** 0.02 ± 0.00 EU/mL) and **9n** (0.02 ± 0.00 EU/mL) and slightly higher for **2n**, **4n**, **8n** (0.16 ± 0.01; 0.31 ± 0.03 0.11 ± 0.00 EU/mL). For other extracts **1n**, **5n**–**7n**, the value was significantly higher and was 0.86 ± 0.01; 1.05 ± 0.00; 0.79 ± 0.08; 0.90 ± 0.06 EU/mL respectively. The statistical analysis has shown a strong correlation between the content of LPS and the ability of extracts to induce production of pro-inflammatory cytokines (Supplementary information).

After the ultrafiltration process, the LAL test was conducted again for extracts at the highest concentration of 50 μg/mL (Fig. 3b). Extracts **1f.**, **4f.**–**7f.** showed lower endotoxin content comparable to native extracts (0.11 ± 0.00; 0.11 ± 0.00, 0.14 ± 0.00; 0.13 ± 0.00; 0.15 ± 0.00 EU/mL respectively), being at the border of applied test analytical sensitivity (0.1 EU/mL). For other extracts, the endotoxin content changed slightly or remained at a similar level as before. The differences between the LAL test for the native and the filtered extract at low LPS content were caused by analytical sensitivity declared by the test manufacturer.

### Evaluation of the mechanism of neutrophil stimulation by tested extracts

**Figure 4:**
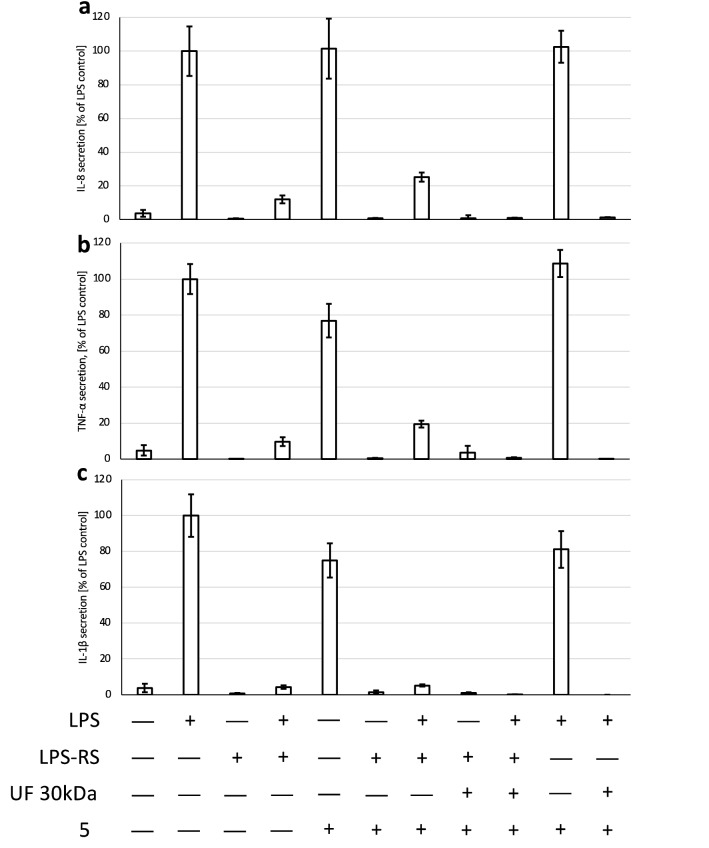
Influence of competitive TLR4 antagonist—LPS-RS (10 μg/mL) on secretion of IL-8 (**a**), TNF-α (**b**), IL-1β (**c**) following neutrophil stimulation by extract **5** (50 μg/mL) and/or LPS (100 ng/mL). Data were expressed as mean ± SEM of three separate experiments conducted in triplicate. The experiments were performed with cells isolated from three independent donors.

To confirm that TLR4 receptor stimulation was responsible for the induction of neutrophil inflammatory response by the tested extracts, LPS-RS—a selective, competitive inhibitor of the TLR4 was used^23^. The pretreatment of the cells with LPS-RS at the concentration of 10 µg/mL almost completely inhibited the secretion of pro-inflammatory cytokines induced by LPS at 100 ng/mL (Fig. 4a–c). LPS-RS was shown in the same manner to prevent the induction of inflammatory response caused by the native extract **5**, what indicates, that the TLR4 stimulation was responsible for its stimulating effect towards neutrophils. Compatible observations were made, when LPS was added to the extract as an internal control. As in previous experiments, ultrafiltration was shown to abolish the pro-inflammatory effect of extract **5**, also when the LPS at 100 ng/mL was added as the internal control before conducting ultrafiltration.

The determined interference of pyrogens implies the necessity of their consideration and removal when in vitro studies include direct addition of plant extracts to the cell culture, what can be obtained by ultrafiltration, which does not affect extract composition.

### Impact of filtration on the stimulatory activity of the extract

**Figure 5:**
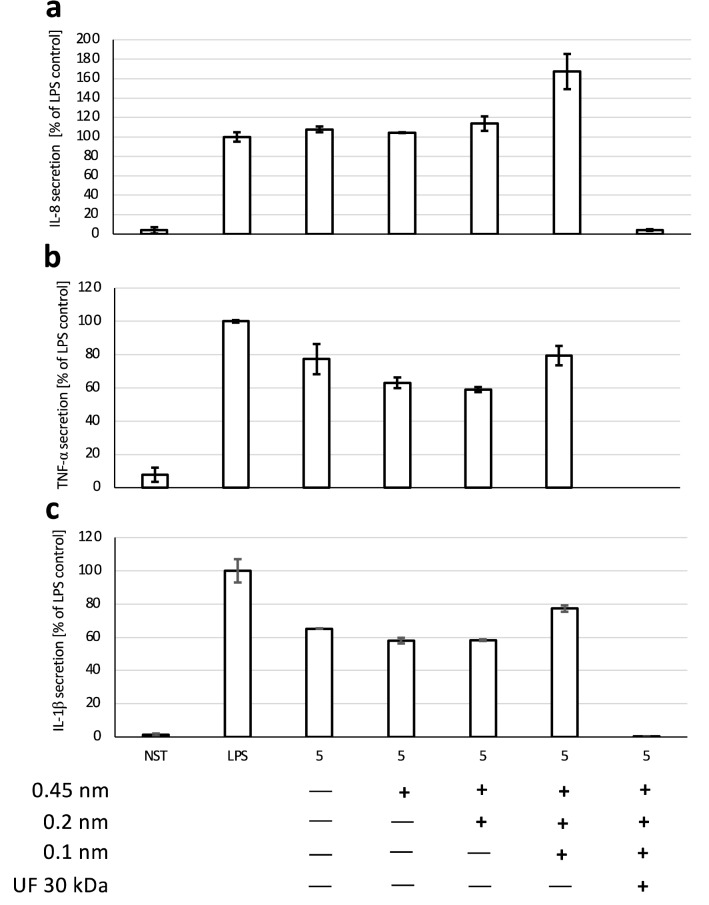
Influence of subsequent filtration steps on extract **5** (50 μg/mL) stimulatory properties towards secretion of IL-8 (**a**), TNF-α (**b**), IL-1β (**c**) by neutrophils. Data were expressed as mean ± SEM of three separate experiments conducted in triplicate. The experiments were performed with cells isolated from three independent donors.

**Figure 6:**
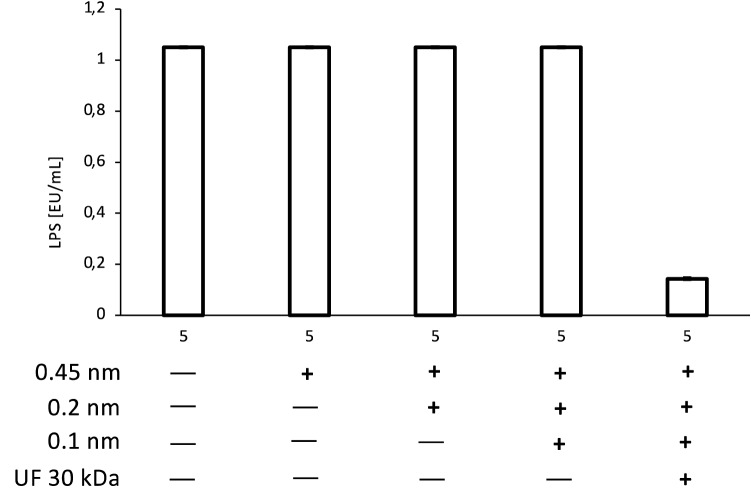
Influence of subsequent filtration steps on pyrogens content in extract **5** at the concentration of 50 μg/mL. Analytical sensitivity: 0.1 EU/mL.

To test whether nanoparticles present in tested extract could be responsible for the induction of inflammatory response of neutrophils, subsequent filtration steps of extract **5** were introduced: 450 nm, 200 nm, 100 nm followed by the ultrafiltration at 30 kDa. Only the ultrafiltration at 30 kDa was capable of removing pyrogenic agents, which confirms that the nanoparticles above 100 nm contained in extract did not contribute to the stimulation of neutrophils (Fig. 5a–c). The conducted LAL test has shown, that only ultrafiltration can result in pyrogens removal, which also excluded the potential interference of nanoparticles with the applied method for LPS determination (Fig. 6).

### Impact of filtration on extract composition

**Figure 7:**
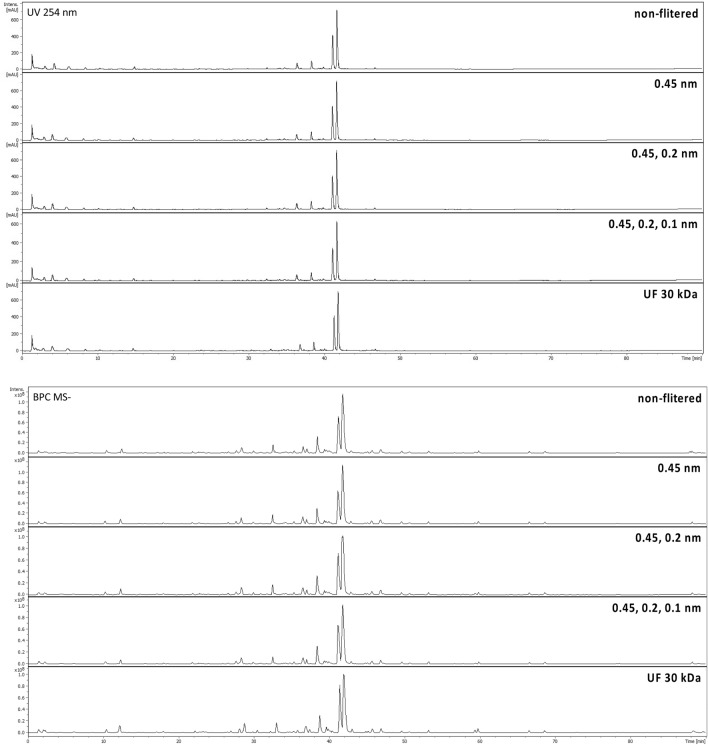
UPLC-DAD-MS analysis of extract 5 filtrated through filters of different size recorded at 254 nm with DAD device (upper) and in ESI—using ion trap mass spectrometer (lower).

In order to determine the changes in specialized metabolites' composition of extract **5** subjected to subsequent filtration, the UHPLC-DAD-MS analysis of its composition was conducted at each step. None of the applied filtration steps, including ultrafiltration at 30 kDa caused changes in metabolite composition profiles of examined extract **5** (Fig. 7).

## Discussion

Many scientific sources indicate the bioactivity of plant extracts confirmed on in vivo and in vitro studies, which support their attributed therapeutic effects in many diseases with the inflammatory background. The discovery of biological activity of plant extract in vitro is a crucial go/no go step for the decision of conducting further studies covering bio-guided fractionation, identification of active principles and mechanisms of action followed by in vivo pharmacological examinations. However, none of the hitherto studies on native plant extracts considered the possibility of the presence of pyrogens, which interference with the applied in vitro models can be of great significance. There is no information about the effect of endotoxins on the change in the biological properties of tested extracts as well as about the undertaken actions leading to their removal^24–26^. We were unable to find any hitherto in vitro bioactivity studies, which would address the issue of pyrogens presence in the tested plant preparations and thus consider their interference with the obtained results. In consequence, there is no protocol specifically introduced to achieve the effective removal of pyrogens from the extracts of plant origin, which could be applied before the introduction of in vitro screening of their biological activities.

The low concentrations of pyrogens, such as lipopolysaccharide, have a less significant meaning in in vivo tests because under physiological conditions of the intact intestinal barrier they are not absorbed from the gastrointestinal tract after oral ingestion. The situation is different when the plant extract is subjected to in vitro tests, which is usually the first step in its bioactivity evaluation. When the tested extract is directly added to the cell culture, the remaining pyrogens get in the contact with the receptors on the cell surface and can significantly interfere with the examined biochemical processes. The obtained results may be disturbed and inadequately referred to the actual activity of the plant extracts and their constituents leading to false positive or false negative results regarding immunomodulatory effects of the tested preparations. As a result, during the in vitro screening studies, some extracts can be considered as ineffective, despite containing active principles as well as some activities can be mistakenly attributed, what can cause inconsistencies during bioactivity guided fractionation and isolation of active principles. The most significant consequence of pyrogens interference with applied in vitro model is the discontinuation of further studies and oversight of constituents with potential pharmacological activity.

The present study clearly shows that applied native plant extracts stimulate the production of cytokines in human primary neutrophils in vitro. The conducted LAL test unambiguously indicated the presence of pyrogens, which concentration correlated with the observed induction of pro-inflammatory cytokines. Extract **5**, as the extract with the highest content of LPS (Fig. 3a) and one of the most strongly stimulating inflammatory response of neutrophils (Fig. 2), was chosen to determine the mechanism standing behind the observed cytokine release stimulation. The experiments using TLR4 agonist confirmed, that the extract contained the LPS, which activated neutrophils in vitro by interaction with this receptor.

We have taken into consideration different strategies to remove the LPS from tested extracts including ultrafiltration, ion-exchange chromatography, gel filtration chromatography, affinity chromatography, sucrose gradient centrifugation and Triton X-114 phase separation^27,28^. Among these, the ultrafiltration appeared to be the method of choice due to the fact, that it is easily accessible and least potent to cause changes in the phytochemical composition of specialized metabolites in the examined extract. Extract **5** as the one with the highest content of LPS was chosen further experiments. Indeed, UHPLC-DAD-MS analysis confirmed, that the filtration at any of the applied filters, including 30 kDa did not cause changes in tested extract composition. It should be noted, that this process causes not only removal of LPS and other pyrogens, but also the molecules of size higher than 30 kDa e.g. polysaccharides, which are not detected by the applied chromatographic method. However, the consequences of removing potentially active molecules of size > 30 kDa are not of a major significance due to their low bioavailability in unchanged form and limited possibility of interaction with inflammatory processes in tissues.

Applied ultrafiltration leads to successful pyrogens removal from extracts what was confirmed both by LAL test and in primary neutrophils in vitro culture. The origin of LPS from bacteria growth in aqueous extracts during the time following the extraction process was excluded by immediate freezing and freeze-drying. What is more, similar experiments were conducted for 70% ethanolic extracts, which results were similar to those obtained for aqueous ones.

Not only pyrogens but also the presence of nanoparticles of plant origin should be considered in in vitro examination of the plant extracts biological activities. Nanoparticles are known for their potential interference with the immune response^29^, thus to exclude their contribution to observed neutrophil stimulation, subsequent filtration steps were introduced, to determine what is the size of particles/molecules which induce cells' inflammatory response. The applied filters in the range 450–100 nm did not remove the stimulating agent, only ultrafiltration using 30 kDa membrane resulted in its complete removal, also when LPS was added to the extract as the internal control. The pyrogenic agent elimination by ultrafiltration was also confirmed using the LAL test, which excluded the potential interference of nanoparticles in the applied method for pyrogens monitoring^30^.

Although the conducted ultrafiltration was shown to effectively remove pyrogens (confirmed by LAL test), some of the extracts were still capable to enhance the production of IL-8, TNF-α and IL-1β by neutrophils. This observation suggests the presence of the principles which act as immunostimulatory agents or have abilities to prime neutrophils but are not pyrogenic. The mechanism standing behind these observations requires however further investigation.

None of the tested extracts, both before and after filtration, showed cytotoxic activity on neutrophils. Before the ultrafiltration, for all extracts, the percentage of viable cells was very high and comparable with LPS control what additionally confirms the presence of pyrogens which are known to inhibit neutrophil apoptosis^21^. Besides, the prevention of apoptosis increased together with the concentration of extracts. After the elimination of endotoxins in the ultrafiltration process, the number of viable cells decreased and reached the level of non-stimulated control.

The results of conducted studies clearly indicate, that LPS contained in plant extracts interferes with in vitro human primary neutrophils model and can lead to significant disturbances of the obtained results regarding their immunomodulatory activity. These observations indicate the necessity of pyrogens removal whenever the in vitro studies include the addition of plant extracts directly to the cell culture in order to avoid false-negative or false-positive results. Ultrafiltration was shown to be an effective strategy for pyrogens removal, without affecting extract composition, which performance was validated by the LAL test and on native primary neutrophils model. Following the ultrafiltration, the tested extracts did not affect the pro-inflammatory functions of neutrophils.

## Methods

### Chemicals

Roscovitine (98% purity), propidium iodide (IP) were purchased from Sigma-Aldrich GmbH (Steinheim, Germany). DMSO was purchased from Chempur (Piekary Śląskie, Poland). LPS (lipopolysaccharide) from *Escherichia coli* O111:B4 was purchased from Merck Millipore (Billerica, MA, USA), ultrapure lipopolysaccharide from *R. sphaeroides* (LPS-RS) (Invivogen, San Diego, CA, USA). Annexin V-FITC, binding buffer, penicillin–streptomycin solution and ELISA sets were purchased from BD (Franklin Lakes, USA). LAL Chromogenic Endotoxin Quantitation Kit was purchased from ThermoFisher Scientific (Waltham, MA, USA). PBS, FBS and RPMI 1640 were purchased from Biowest (Nuaillé, France). Pancoll was purchased from Biotech (Aidenbach, Germany). Ultra-pure water was produced with Merck Millipore Simplicity UV system. Urolithin A was obtained in-house^31^. The identity was confirmed by NMR and MS spectra.

### Preparation of plant extracts

*Filipendulae ulmariae herba* (**1**) batch no. 506.2016, *Sambuci flos* (**2**) batch no. 278.2016, *Violae tricoloris herba* (**5**) batch no. 506.2016, *Ononidis radix* (**7**) serial no. 503.2017 and *Phaseoli pericarpium* (**8**) batch no. 1221.2018 were purchased from Kawon. *Betulae folium* (**3**) serial no. 1086, *Polygoni avicularis herba* (**4**) batch no. 1096 and *Hernariae herba* (**6**) batch no. 1096 were purchased from Flos. *Petroselini radix* (**9**) serial no. EH/2018-196 was purchased from EkoHerba. Plant materials were identified based on the microscopic evaluation of the powdered sample according to the monograph in European Pharmacopeia 9.0. For those materials that no monograph is available the comparison with suitable literature was performed^32^. If needed the additional authentication by TLC profiling was performed. Voucher specimens of each plant materials are deposited in the Herbarium of the Department of Pharmacognosy and Molecular Basis of Phytotherapy, Medical University of Warsaw.

Plant materials were extracted twice with boiling water with a material:solvent ratio (*m*/*v*) as follows: 1:16 and 1:10. Obtained infusions were immediately filtered and freeze-dried^33^. Ethanolic extracts were obtained by sonication (for 15 min) using 70% ethanol at room temperature. The extraction was repeated twice. The organic solvent was evaporated under the vacuum at 40 °C. The water residue was freeze-dried. Dry extracts were dissolved in mixture of DMSO:water (v/v, 1:1) to prepare 20 mg/mL stock solution.

### Filtration of stock solutions

In order to determine whether nanoparticles or high molecular compounds are responsible for the interference, the chosen extract was subjected to three steps of filtration. The stock solution of **5** was filtrated through syringe PTFE (Whatman, Little Chalfont, UK) 13 mm filters with decreasing pore size (450 nm, 200 nm and 100 nm). After each step filtrate was stored and used for bioassays and chromatographic determination.

Removal of high molecular pyrogens from extracts was performed for all samples using ultrafiltration process^34^. For this purpose, were used Microsep™ Advance Centrifugal Devices (Pall Corporation, New York, United States). The extracts were centrifuged twice, first using filters with cut-off value 100 kDa, second 30 kDa.

### Evaluation of endotoxins content in extracts

The solutions of all extracts at a concentration of 50 µg/mL in endotoxin-free water were used to evaluation of endotoxins content by LAL test according to the manufacturer’s instructions. Analytical sensitivity: 0.1 EU/mL.

### Isolation of human neutrophils

The buffy coats were commercially obtained from healthy human donors (only men below 35 years old) from Warsaw Blood Donation Centre. Donors declared that they are non-smokers and not taking any medications. Anonymous buffy coats from healthy blood bank donors were only used if donors had consented scientific use of blood products. Donors were confirmed to be healthy; all routine laboratory test was carried out and showed values within the normal range. The study conformed to the principles of the Declaration of Helsinki and experiments were performed following guidelines and regulations of Ethics Committee of the Medical University of Warsaw. Neutrophils were isolated using dextran sedimentation and centrifugation in a Pancoll (1.077) gradient (1500 rpm, 4 °C). Erythrocytes were removed by hypotonic lysis. The purity of the neutrophils preparation was over 97%. After isolation cells were suspended in a medium (RPMI 1640) and were maintained at 4 °C before use^15,35^.

### Incubation of neutrophils with plant extracts

After isolation, the cells suspension in RPMI 1640 (with stable glutamine, 25 mM HEPES and with the addition of 10% FBS and 1% penicillin–streptomycin solution) was seeded on a 96-wells plate at the concentration 2 × 10^6^ cells/mL. To appropriate wells were added tested extracts at final concentrations in well 6.25, 12.5, 25 or 50 µM. Cells with extracts were incubated in 24 h at 37 °C with 5% CO_2_.

### Evaluation of neutrophils’ viability and apoptosis

The viability and apoptosis of neutrophils was determined by staining with propidium iodide (PI) and Annexin V^35^. After 24 h incubation cells were centrifuged (2000 rpm, 10 min, 4 °C), the supernatants were collected, and cells were washed twice with 500 µL of PBS. Then, the neutrophils were resuspended in binding buffer and suspension was taken to another 96-well plate to obtain a concentration of 0.5 × 10^6^ cells/well (4 × dilution). In the next step, the staining solution of Annexin (5 µL/mL) and IP (0.5 µg/mL) in binding buffer was prepared and added to each well. After 15 min incubation at room temperature in darkness the cells were analysed by flow cytometry (FACSCalibur) within 1 h after labelling and data from 10,000 events were recorded in the gate. Before each experiment compensation was performed and quadrants have been set. Urolithin at the concentration 5, 10 and 40 µM and roscovitine at 40 μM were used as a positive control—apoptosis inducer. LPS at the concentration of 100 ng/mL was applied as a positive control—prevention of apoptosis.

### Evaluation of IL-8, TNF-α and IL-1β production

The collected supernatants from stimulated and non-stimulated neutrophils were used to evaluate cytokine production (IL-8, TNF-α and IL-1β) using enzyme-linked immunosorbent assay (ELISA) according to the manufacturer’s instructions. In the last step of the test, absorbance at 450 and 570 nm was read using a plate reader (BioTek). Urolithin A at the concentration 5, 10 and 40 µM and roscovitine at 40 μM were used as a pure compounds negative controls^36,37^.

### Phytochemical comparison of extracts with UHPLC-DAD-MS method

The UHPLC analysis was performed using Ultimate 3000 series system (Dionex, Idstein, Germany) coupled with Amazon SL ion trap mass spectrometer (Bruker Daltonik GmbH, Bremen, Germany). The separation was carried out with Kinetex XB-C_18_ column (100 mm × 2.1 mm × 1.7 µm), Phenomenex (Torrance, CA, USA). The oven temperature was set at 25 °C. The column was eluted with A (0.1% HCOOH in H2O) and B (0.1% HCOOH in MeCN) with a two-step gradient as follows: 0 min 1%B, 60 min 26%B and finally 90 min 95%B. The flow rate was set to 0.300 mL/min. Five µL of each sample (10 mg/mL) was injected. UV–Vis spectra were recorded in the range of 200–450 nm. Chromatograms were acquired at 254 nm. The ion trap Amazon SL mass spectrometer was operated with the ESI interface. The parameters for ESI source were set as follows: nebulizer pressure 40 psi; dry gas flow 9 L/min; dry temperature 300 °C; and capillary voltage 4.5 kV. The analysis was carried out using a scan from m/z 70–2200. Chromatograms as base peak ion current were recorded in negative mode only.

### Statistical analysis

The results were expressed as a mean ± SEM. Statistical significance of differences between means was calculated using one-way analysis of variance (ANOVA) with Tukey’s post hoc test. Statistical significance was set at P < 0.05. All analysis were calculated using Statistica 10.0 (StatSoft) or Excel 2016 (Microsoft Corporation).

## Supplementary Information

Supplementary Information.

## Abbreviations

1f.…9f.: Extracts after ultrafiltration
1n…9n: Non-filtered extracts
CDK: Cyclin-dependent kinase
DAMPS: Damage-associated molecular patterns
FBS: Fetal bovine serum
LAL: Limulus amebocyte lysate
LPS: Lipopolysaccharide
LPS-RS: Ultrapure lipopolysaccharide from *Rhodobacter sphaeroides*
NETs: Neutrophil extracellular traps
PAMPs: Pathogen-associated molecular patterns
PI: Propidium iodide
rsc: Roscovitine
TLR: Toll-like receptor
uroA: Urolithin A
