# Supplementary material for: High molecular pyrogens present in plant extracts interfere with examinations of their immunomodulatory properties in vitro
Source: Sci Rep. 2021 Jan 12;11:799. doi: 10.1038/s41598-020-79579-2 (PMC7804927; doi:10.1038/s41598-020-79579-2)
Supplement: Supplementary file 1 — Supplementary Information. [file 41598_2020_79579_MOESM1_ESM.pdf]

Supplementary material

High molecular pyrogens present in plant extracts interfere with examinations of their immunomodulatory properties *in vitro*.

Aleksandra Kruk[1,2], Jakub P. Piwowarski[*1,2], Karolina A. Pawłowska[1,2], Dominik Popowski[1,2], Sebastian Granica[1,2]

[1] Department of Pharmacognosy and Molecular Basis of Phytotherapy, Centre for Preclinical Studies, Faculty of Pharmacy with the Laboratory Medicine Division, Medical University of Warsaw, ul. Banacha 1, 02-097 Warsaw, Poland

[2] Centre for Preclinical Studies, Medical University of Warsaw, ul. Banacha 1b, 02-097 Warsaw, Poland

[*]Corresponding author

Jakub P. Piwowarski, Department of Pharmacognosy and Molecular Basis of Phytotherapy, Faculty of Pharmacy, Medical University of Warsaw, Banacha 1, Warsaw 02-097, Poland, tel. Tel.: +48225720953; fax: +48225720985

E-mail address: jakub.piwowarski@wum.edu.pl (J.P. Piwowarski)

Appendix A. Supplementary data

The graphs (Figure S1) and Table S1 show the correlation analysis lipopolysaccharide content of the extracts and/between IL-8, TNF-α and IL-1β production by neutrophils. The analysis concerns non-stimulated extracts.

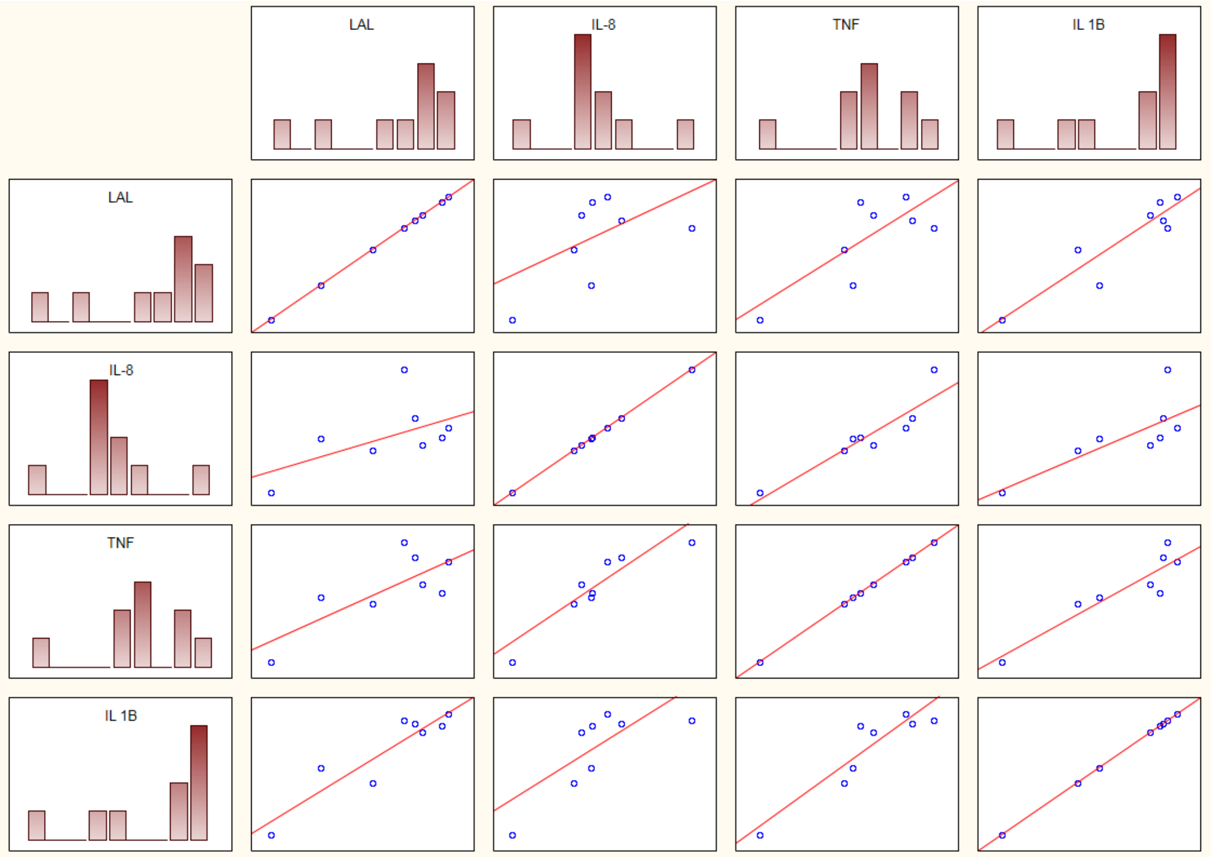

Figure S1. Graphic presentation of correlation analysis.

Table S1. The correlation coefficients.

	LAL	IL-8	TNF-α	IL-1β
LAL		0,54	0,77	0,92
IL-8	0,54		0,91	0,75
TNF-α	0,77	0,91		0,92
IL-1β	0,92	0,75	0,92	

Based on the analysis, it can be concluded that there was a moderate relationship between the LPS content in the 50 μg/mL extracts and their influence on cytokine production for IL-8 (correlation coefficient 0.54), strong for TNF-α (0.77) and very strong for IL-1β (0.92). Between cytokines, strong relationship was observed for IL-8 and IL-1β (0.75), while a very strong for TNF-α and IL-8 (0.91) or TNF-α and IL-1β (0.92). All correlation coefficients were statistically significant, except coefficient between LPS content and IL-8.
